# A Sisterhood of Hope: How China’s Transgender Sex Workers Cope with Intimate Partner Violence

**DOI:** 10.3390/ijerph17217959

**Published:** 2020-10-29

**Authors:** Eileen Yuk-ha Tsang

**Affiliations:** Department of Social and Behavioral Sciences, City University of Hong Kong, Hong Kong, China; eileen@cityu.edu.hk

**Keywords:** transgender sex workers, intimate partner violence, China, informal networks, sisterhood, commercial sex work

## Abstract

Transgender sex workers (TSWs/TSW) face considerable challenges that affect their mental health and make their situations more vulnerable and precarious. TSWs often experience violence from clients, police, and others, but it is estimated that 50% of these acts of violence are at the hands of their intimate partners. The marginalization of TSWs is fueled by abuse through isolation and shaming which prevents them from seeking help through formal channels like police or counselling services. There is limited research on intimate partner violence (IPV) involving transgender sex workers (biologically male at birth who transition to women) and their partners who are typically heterosexual/bisexual men. In China, stigmatization, homophobia, heterosexism, and transphobia structurally disadvantage TSWs and this power structure tacitly supports violence and abuse against them. To survive, TSWs rely on informal networks with their ‘sisters’ for advice and emotional support which is more effective at combatting IPV than criminal justice or social policy efforts. Ethnographic data from in-depth interviews with 25 TSWs provide insight about IPV and how informal social support is a protective factor that helps them cope with routine acts of violence. The findings identify the importance of the ‘sisterhood’ and how it protects and helps TSWs manage their physical and mental health.

## 1. Introduction

A significant social issue in China involves a group of individuals who exist on the margins of society. They are shunned and rarely discussed or even acknowledged to exist. In the West they are referred to as ‘transgender’ and face inordinate levels of stigma and discrimination. The term ‘transgender’ applies to people whose identity is mismatched with their biological gender, resulting in gender-reassignment procedures. Mismatches may occur with either gender, but in this article particular attention has been placed upon those born biologically male who identify themselves as female.

A transgender person is an individual assigned masculine or male sex at birth, but whose identity over time emerges to be feminine, regardless of genitalia, behavior, or plans to alter their bodies [1]. People who identify outside of the gender binary are often referred to as “non-binary.” Non-binary gender identity can include identifying as neither male nor female, both male and female or as different genders at different times [2] Transgenders fit into the non-binary gender categories.

Globally, the overall number of transgenders is increasing. It is estimated that 9.2% out of every 100,000 transgenders have received or requested gender affirmation surgery or transgender hormone therapy; 6.8% out of every 100,000 have received transgender-specific diagnoses; and 355 out of every 100,000identify themselves as transgender [3]. The number suffering abuse and violence is also increasing. A study from Massachusetts in the U.S. surveyed 1600 people and found transgender respondents reported lifetime physical abuse rates by a partner of 34.6%, versus 14.0% for gay or lesbian individuals [4]. Another study reported up to 50% of violence experienced by transgenders comes from their intimate sex partners [5]. The rising number of TSWs in China raises significant public health questions. For example, TSWs are 49 times more likely to acquire HIV than all adults of reproductive age, and the rate of HIV infection among TSWs is 27.3%, nine times higher than female sex workers, and three times higher than gay sex workers [6,7].

TSWs are also vulnerable to the vastly under-researched area of Intimate Partner Violence (IPV). To date, there are no official estimates regarding how many transgenders exist in China, but one rough estimate is 400,000 [8]. Indeed, transgender-specific data collection, HIV programming and outreach is almost non-existent in China. All over the world, transgenders disproportionately experience human rights violations in the form of stigma, discrimination, and violence [9,10,11,12].

IPV must be addressed because of the relationship between issues of employment, infection with HIV, and emotional stability. Rates of self-harm, suicide, violence, and bullying are high and linked with IPV [6]. TSWs are perhaps the most precarious and vulnerable group in China’s commercial sex-scapes, not least because of the difficulty in finding compatible sex partners who do not treat them like freaks or novelties. Like TSWs in many other countries, Chinese judicial law does not recognize transgender identity; the term is not even mentioned which implies the category does not even exist; there is no mention of gender other than traditional ‘male’ or ‘female’. As such, issues like IPV remain largely unexplored, undeveloped, and unmentioned in today’s China [13].

This article argues that transgender is a non-binary category of individualization which needs protections in order to safeguard and improve the living conditions of this vulnerable community. As such, informal networks may be better mechanisms for fighting IPV than the policy making and criminal justice systems. In this study, 25 TSWs were interviewed about the growing stigma attached to IPV, health risks such as contracting STIs, and occupational hazards such as being arrested and given detention. The interviewed 25 TSWs all experience at least one form of IPV, including sexual assaults, physical violence, verbal assaults, being financially ripped off, stalking and extortion. This article seeks to: (1) fill in the research gap using queer criminology by emphasizing the importance of emotional support from other transgender sex workers; (2) to examine TSWs self-reliance strategies, comparing the perceived effectiveness of those efforts with formal state-sanctioned migrant assistance programs; (3) to expand Western scholarship on intimate partner violence and provide empirical data from a non-Western country.

## 2. Conceptual Framework

Studies typically identify how men use violence as an extension of patriarchy against female victims [14], or look at transgender as a general population [15,16,17,18,19,20,21,22]. The literature on transgender has considered HIV, sexual and mental health issues in China [23,24,25]. Much of the literature affirms that heterosexual men are often the primary agents of aggression and victimizing behaviors against heterosexual women. Men still routinely enact hegemonic masculinity [26] and transgenders are depicted as victims of patriarchy [27,28] against heterosexual women. Minority groups like lesbian women, gay men, transgenders, and bisexuals are at the bottom of the gender hierarchy, which perpetuates men’s privileges at the top. This, in turn, disadvantages transgenders who do not perform gender heteronormativity, and are often deprived of political, social and cultural power.

Some literature also uses the Foucauldian perspective to assert that power is not just structural or held by a group of individuals, but rather emerges from the discourse between individuals [29]. For transgender individuals, this power lies behind the cisgendered discourse that shapes structural responses to IPV [30]. Therefore, IPV is not solely a result of a patriarchal power structure, but rather a consequence of structurally informed discourses that not only marginalize transgenders but also create distinct realities across race, class, sexual orientation, and gender identity. However, the literature on IPV may not be strictly applicable to the phenomenon of IPV toward TSWs. TSWs are not within the logical framework of heterosexuality, heterosexism, and the normalization of heterosexuality, or heteronormativity. Transgenders do not fit neatly into the gender binary of being only either man or woman.

Queer criminology has been used to explain IPV against transgenders in Western countries like the United States [27]. Queer criminology refers to “exploring the manifestations of transphobia and homophobia in the realm of crime and criminal justice” [31,32]. However, Western queer criminology relies on the criminal legal system to improve the precarious situation of transgenders, but China’s criminal justice and social policies are not inclined to help TSWs. Instead, TSWs must rely on informal support networks to help them cope with IPV.

### Holistic Approach—Queer Criminology and the Importance of Informal Sectors

Queer criminology treats transgender as a fluid, dynamic and non-binary category, but does not agree that criminal justice and social policies can help TSWs overcome stigma, as in Western countries. Rather, self-help programs can help TSWs solve their own problems. Guadalupe-Dia [27] noted that self-help programs encouraging the transgender population to seek help from their friends or NGOs helped them solve IPV-related trauma related to depression and emotional frustration. Studies of female sex workers (FSWs) in Brazil [33], Turkey [34], India [35], and Swaziland [36] show that trust, solidarity, and social cohesion within the community of FSWs increased condom negotiation with sexual partners resulting in higher rates of condom use. In China, Zheng (2008) [37] found bar hostess’ support networks in China were enabled by blood relationships, common background or birthplace, and mutual benefits for female sex workers.

Therefore, informal social support coming from sisterhood and social cohesion seems to moderate the relationship between IPV and symptoms of depression, even though these females also suffered from police harassment [1] and different forms of IPV from their sex partners [27]. Informal networks would appear to be more useful to help TSWs overcome the effects of IPV in China. In the face of healthcare barriers, TSWs may turn to their peers for social support and assistance to address that unmet need. The 25 TSWs interviewed reported that their sisterhood—other TSWs—provided emotional support, physical safety and protection against their partner, and even against the police.

## 3. Methodology

The fieldwork took place in Tianjin, North China. Ethnographic field notes were obtained through several excursions to North China over a three-year period, from May 2016 to August 2019. During winter breaks and summer months the author worked in a bar employing several TSWs. The time spent there was roughly three–four months per year, totaling nine–ten months of field work data collection. The trips ranged from relatively short (one week) to long (four months), comprising seven to eight trips per year to the sites of interest. This approach was better suited to academic employment obligations and more feasible than extended immersive on-site fieldwork. The fieldwork data collected should be sufficiently ‘thick’ to enable analysis of the complex web of client–worker relationships. The TSWs knew that the author was a full-time researcher but not a full-time bartender, therefore, they could maintain a professional relationship. Much of the data collection came from two primary excursions, but with seven or eight trips per year to North China, using ethnography and in-depth interviews. The first excursion occurred between May 2016 and December 2018 when the author worked as a non-paid bartender in one of the high-end gay bars in Tianjin from May 2016 to August 2018. She conducted interviews with ten TSWs who had experienced IPV out of a total of 51 male sex workers who worked in the bar. The second excursion for data collection in China gathered data through twelve LGBT non-governmental organizations (NGOs) in North East China from September 2018 to August 2019. The NGO in Hong Kong helped with initial connections, and snowball sampling helped recruit additional respondents. After this second excursion, the author interviewed 49 TSWs, of whom 15 respondents admitted they had experience with IPV. In the two excursions, a total of 25 respondents were interviewed who suffered from IPV, ranging from 23 to 48 years of age.

Data comprised recorded interviews, in situ note-taking, and post-event field notes. Prior to any recorded interviews, all the respondents signed consent forms. Respondents were given a copy of the author’s business card and contact details and reminded that they could withdraw without prejudice at any stage in the project. To safeguard the rights of the informants, the author verified her credentials and was neither affiliated with the police nor the government. The informants were fully assured of confidentiality and anonymity as the author used only their current ages and assigned pseudonyms. Personal information such as official identification numbers or date of birth were not collected. Protecting informants’ privacy was of paramount concern due to the sensitive nature of the data.

Criteria for the selection of interview participants were: (1) self-identification as TSWs; (2) aged over 18 years; (3) had experienced at least one form of IPV (sexual assault) from their former or current intimate sex partners and were willing to talk about it. All of the participants characterized their intimate relationships as romantic, sexual, and emotional. The duration of these relationships ranged from 30 days to four years (48 months).

The interviews were conducted one-on-one, in person, in private locations. Interviews generally ran around one hour in length. Topics included how the informants became TSWs, some family history or life story as to how they knew they were transgender, how they connected with their sex partners, episodes of IPV and how they reacted, their perceptions about being bullied, and preventive measures they took to help them avoid IPV. Additional questions examined circumstances when leaving their village and traveling to the city, and whether they originally intended to become sex workers. Details about how they entered sex work included critical moments when they made key decisions. Respondents were also asked about the role of drugs, sexual activities, risk of HIV, and circumstances surrounding sex-change, along with details about sex-change in China (where to go, how to get there) and the discrimination and resistance they experienced since undergoing surgery. Finally, all respondents were asked for details about their intimate relationships—how they met, how they progressed, roles and behaviors, and particularly the elements that constitute abuse—sexual, physical, verbal, stalking and extortion.

All the interviews were recorded, transcribed, and analysed with the guidance of grounded theory [38]. The transcripts were translated by the interviewer into English and NVivo 11.0 software was used for coding and analysis. Preliminary coding began by reading and rereading five transcripts. A codebook was then developed to cover themes drawn from the interview guides, as well as new themes that emerged during the coding process. The ethical approval of this research protocol came from the author’s Institutional Review Board (reference number: 3-9-202003-04).

## 4. Results

### 4.1. Meeting Meiha

All of the 25 TSWs had suffered from sexual assaults, and over 15 out of 25 TSWs had suffered from more than one type of IPV like physical assaults, verbal assaults, being financially ripped off, stalking and extortion. Physically, all 25 TSWs had already undergone artificial breast surgery. Twenty-one had this work performed in Thailand and the other four in China. However, none of them had yet undergone vaginoplasty surgery. All of them came from rural villages in northeast China, and 19 said they had followed social norms and at one time were married to women. At the time of interview, many still had not divorced their heterosexual wives. Instead they had simply moved away to work in the city while the wife stayed in the country village. The remaining six were single. All the respondents admitted difficulty in finding intimate partners, and this explained why most of them did not fight back as they are too scared of being alone.

Intimate partner relationships rarely occur through sex work; only two admitted their partners had been former clients. A total of 21 out of the 25 TSWs regularly use drugs or alcohol and said sex does not include condoms. Only four said their partners occasionally used condoms. The reasons given for drug use included pleasure and escape. Drugs were credited with helping them deal with the stress of being a TSW in Chinese society [39,40,41].

The author met Meiha (48) in summer 2018. Her experience encapsulated the forms of IPV, her struggles with her intimate sex partners, seeking help from her sisters, and antagonistic conflicts with the police and government officers. She has been a TSW for more than six years in a theatre where she performed Chinese opera wearing heavy makeup. She was tall, slim, and looked delicate, even fragile. She was an outstanding performer and often received generous tips upwards of 1000 yuan (US $150). She is older than most TSWs, but she keeps herself physically fit and her skin moisturized and unblemished. She knew she wanted to be a woman at age 15. When she was 22 years old, Meiha fulfilled the filial obligation in China where a man marries a woman and has children. The two of them produced one son and one daughter. With only a primary school education, Meiha worked in the village at a factory for 20 years, but once both children became adults and the elder son finished college, Meiha came out to his family as gay. They reacted by calling him a monster and asked him to leave the village.

So Meiha moved to the city, and in early 2014 successfully transitioned via breast implantation surgery. Meiha said her only path then was to become a freelance TSW. She installed the gay dating app *Blued* onto her phone and began meeting interested men. She soon met her partner, whom she described as strong and tall, and they moved in together in December 2014.

Meiha said her partner is the only one she has ever kissed in public. They would walk publicly hand-in-hand despite hearing derogatory comments from people around them. But Meiha did not care. However, she soon discovered that her partner was also very controlling, and the relationship began a downward spiral. As we spoke after her opera performance that night, she suggested we go have a drink in a nearby bar.

E: How did you discover your partner’s true character?

Meiha: He always wanted more money to buy drugs and alcohol. But when he had them, they made him frantic and unstable. Once, he demanded I find more clients to make more money and I refused. He erupted like a volcano. He yelled at me, scolded me, then began beating me.

E: Did you fight back?

Meiha: Yes, I did. But the more I fought, the angrier he became. He damaged my apartment, smashed my lamps, my TV, the furniture, and he even threatened me with a knife. Usually when we fought I would tolerate him. It is difficult for me to find a boyfriend, much less a life partner. That night we fought, I finally told him he should move out. He was angry, but he left without incident. The next day I was going to change the lock, but he came to my apartment and said he was there to pack his belongings. I was not paying attention that he had brought a small knife. He walked up to me in our kitchen and I felt pain in my arm. He had stabbed me! I screamed and pushed him, then ran away. I left the building and hid in an alley. I called a friend to take me to hospital. I did not dare to call the police. They would just make fun of me.

E: What happened at the hospital? What did you tell them?

Meiha: I had to pay for treatment from my own pocket because I had no health insurance from the government. I admitted that my partner attacked me, and the hospital insisted I report it to the police. But I refused. Being transgender, my identity is not approved… So I stayed silent and afterward, I moved to another city, and changed my name, address, and mobile phone number. Hah! He still found me using that damn dating app. I still don’t have medical insurance to support my therapy and medication, and I still have to pay from my pocket to see a doctor regularly. So, I write a journal and talk to my sisters online…I’m glad they listen to me.

E: Why didn’t you leave him sooner? Find another partner. Why tolerate him?

Meiha: I liked him. I was hoping he would change and did not want to drive him away. In the beginning, I tolerated him and sacrificed a lot. It is not easy for someone like me to find a partner.

E: Why didn’t you call the police?

Meiha: Because I am transgender, I avoid attention from the Chinese police and government. Calling the police brings more trouble. I was caught having sex in the park and they put me in a detention center. I don’t want the police to bother me. They just make fun of me.…

E: What kind of help do you get from the criminal justice system? What social policies help you?

Meiha: No, nothing. I think other TSWs who have similar experiences help me. I use WeChat to confide with one particular sister about problems with my partner. Sometimes, I get tired of playing the arrest game with the police. They catch me in the park or in a street alley. I then call other sisters nearby on WeChat to come rescue me. Sometimes, they go get the gangsters, and for some extra money they come help me. We will all go to the police station together so we have more bargaining power. Sometimes, we even call a journalist to try and draw more attention. The police don’t bully so much when there’s a lot of attention.

Meiha’s case documents the conflicted professional life experienced by many TSWs. Against professional stigma and fighting with police, all 25 TSWs interviewed admitted they also regularly struggled at home with IPV. It is significant that each TSW said their partner was a heterosexual man who had expressed interest in them specifically because they were transsexual. There were 13 TSWs who said that their partners had difficulty holding a job or finding work. Reasons given by the TSWs included low pay, poor work conditions, lack of professional skill, or sometimes the catch-all term ‘laziness’. The other six partners were employed but working in low-wage occupations such as bartender, salesclerk, factory worker, and taxi driver. All 25 TSWs said they were the breadwinners, providing daily living expenses, food, and housing for their partners. Only five of the TSWs said they fought back against the IPV. The others admitted that they did not even try to retaliate because they feared they would lose their relationship and be alone.

### 4.2. Different Forms of Intimate Partner Violence

#### 4.2.1. Sexual Assaults

Sexual assaults are the most commonly discussed form of IPV among the TSWs. The 25 TSWs spoke at length about sexual assaults from their partners. For example, Yee is a 24-year-old TSW who lived with her intimate sex partner for one year before they broke up. When interviewed in the conference room of an NGO, Yee did not wear makeup and was dressed in jeans and a T-shirt. With her naturally straight, long black hair and artificial breasts she looked like a typical young Chinese woman [42].

Yee said that in the beginning her partner often spoke of his infatuation with her artificial breasts and his curious excitement that they both had male organs. For a while they seemed compatible to live with each other and she willingly accommodated her partner’s sex drive. But after the first month, he began collecting sex toys and fantasy costumes. The sex became progressively more extreme and less pleasurable. Yee did not know exactly when it turned abusive:

… His favorite thing became using a dildo on me that pulses and throbs to music. Even now, he inserts it and then I have to do a striptease dance for him. He says it helps arouse him, and he masturbates as I dance. In bed he treats me like I’m a toy for his pleasure. Sometimes he has me blow into his anus with a straw, and sometimes he wants me to urinate as I give him a blowjob. Once I ended up vomiting from the filth and had to go to the hospital. I told him many times I don’t like these things but he doesn’t listen to me… One night, I refused his requests and he shouted at me: “Who do you think you are? You are only a she-male, a monster! You are acting like a princess⸺What a joke! huh?!” He then beat me with the dildo and used it on me again...

Although they broke up, the IPV has continued and she remains conflicted about resolving the relationship. Her ex-partner works in a nearby mobile phone factory and still regularly visits her apartment where he forces himself sexually upon her. Yee does not recognize that she is being serially raped. Instead, she dismissed the assaults with a shrug, saying “he is always hungry for sex.” Remarkably, Yee shifted in mid-sentence from how he still bullied her and pushed her to get vaginoplasty, to the neutral topic of the procedure itself. Yee went on at great length about how the procedure was too expensive, and that if she could raise the money she would prefer to have it done in Thailand because, although the procedure is cheaper in China, she worried whether it would be done correctly.

Another TSW, Lily (28) shared her experiences with “chem fun parties” (Chinese: *bunfun paidui*) sex parties involving up to 30 men and women lasting several days which take place at private houses or luxury hotel suites and are catered with drugs, alcohol, and food)…

… Sometimes my lover beats me and slaps me because he says he loves to play the SM! He will tie me to the ceiling with a red rope and drip candle wax all over my shoulders, my breasts, my thighs. Sometimes he uses a whip that leaves marks on my ass. If I refuse, he just hits me and slaps me some more… He likes a big finish on my face, my hair and my body which I have to clean up afterward.

Ruijia (39) had the same experience of sexual assaults from her partner. She says,

I remembered he played with my sexual organs like he was studying them, poking, prodding, squeezing my breasts and my penis. He acted like a stranger, a doctor taking measurements. There was no emotion or feeling and I felt hurt and humiliated. Afterward he said he wanted to invite girls to our apartment to have group sex⸺even a dog if possible! In the end he backed down, but I don’t know…Some men and women want to cosplay like they become a cross-dressing Queen or King… My partner will also mix *maotai* (80–100 proof clear Chinese rice liquor) with the drugs, but it often makes him abusive. He gets loud and starts saying whatever he wants, like calling me a filthy dirty whore…

#### 4.2.2. Physical Assaults

Fan (24) is a TSW who lived with her boyfriend for more than two years. They met each other through a gay dating App [43]. Her sex partner was a factory worker who hated his job. He kept changing jobs and finally stopped working altogether. He said he wanted to be a “kept man”, financially supported by Fan. Fan says:

After taking the drugs, my partner can be physically brutal. We fight when he is like this, yelling at each other. He is so critical of what I do and who I am. Occasionally it gets so bad he hits me. I did leave him… but we are still in the midst of resolving this mess… his stuff is still in my apartment. I need to see a doctor regularly for my depression.

The author met Juan (32) in a high-end bar. She was wearing an elegant tight dress that accentuated her slim figure and highlighted her long black hair. She was the picture of femininity. As we spoke, Juan talked about her stage performance and her love of music and dancing. Her accomplishments are noteworthy; she has regularly appeared on nationally televised variety shows. Officials routinely invite her to perform for audiences in rural and poor areas. She bragged about how her dancing captivated ethnic minority audiences in Xinjiang and Tibet. Her typical outfit includes elaborate make-up and full-length traditional costume that leaves no skin showing from the neck down.

Juan (32) said China is still very conservative, despite having “bid farewell to the concubine.” Juan said it is still frowned upon for men to hold hands, let alone kiss, in public. She and her partner, a DiDi driver (taxi driver), had a seven-day honeymoon period when they first got together, holding hands and kissing in secret whenever they could. But romance was replaced by practical aspects and conflicts emerged,

One day, we had an argument over finances, like who pays for rent and who pays for food. I had been paying the rent and all the other monthly bills. He said his slim salary could not even buy him lunch and keep gas in his car. He thinks my salary is so easy to make and so much higher. Our argument became a fight and he ended up cutting me with a knife. I have not forgiven him and our hostility is still unresolved…

Faho (35) had a similarly story of violent abuse. She says,

… My partner hid a fruit knife in his sleeves. When I wasn’t paying attention he suddenly stabbed me and punched me, breaking my ribs. I had to stay in a hospital for six months. The attack that day was the result of a series of fights we were having. Still, I forgave him…The hospital asked me what happened. I said I hurt myself…

#### 4.2.3. Verbal Assaults

The 25 TSWs mostly discussed verbal assaults within the context of body shaming. The partners openly disparage the bodies of TSWs, mocking, teasing, even openly laughing at them. Not surprisingly, verbal assaults can escalate into physical attack once drugs or alcohol are introduced into the situation. Yoyo is a 44-year-old transgender woman who completed gender reassignment breast surgery. Afterwards, she met her boyfriend in 2017 through an online app and they conversed for two days before she worked up the courage to meet him in a bar. Yoyo married a woman several years ago and they had two sons. The sons, now 18 and 20 years old, study at a university in China. Yoyo said her wife knows she prefers men but is ignorant about her being transgender. When Yoyo came to Tianjin in 2015, she initially engaged in cross-dressing. She bought wigs, dresses, high heels, and learned to use makeup. She met her first and only partner at the club where she performed, a bartender who worked there. They quickly moved in together. Her partner always complained that the working hours were too long for the meager wages. Although passion disappeared after the first two months, they stayed together for four years. Yoyo admitted that they stopped having sex a long time before they eventually broke up.

We felt bored to each other. He asked for sex occasionally after our honeymoon period, but I steadfastly refused him because he kept insisting I get vagina surgery. Each time I refused, we would argue and eventually he would laugh at me… Recently we had a huge fight that ended everything. He was very depressed one night and started drinking. He was soon touching me and then started undressing me. I told him to stop. He pulled out his phone, pointed at my sexual organs and started laughing as he took photos. I was so shocked and speechless I could not move. Finally, I told him that he is a scoundrel and that we are over… I hate him. That night he said I am so desperate to get a man, but no man would want me! He said even though he is a bully, I should be grateful. He is allowed to conquer and control me because he is a heterosexual man! I am only a transgender woman, a double loser, neither man nor woman. I am a monster!

Jackie (32) has been a TSW for four years living with her partner for six months. Jackie found that she and her partner were not compatible after the third month of living together and fought almost every day. But one day in 2010, her partner was in a car accident and, despite therapy and rehabilitation, remains wheelchair-bound. Jackie said her partner could be very hot-tempered, but Jackie loves him a lot and promised to take care of him. Jackie discovered her partner was emotionally and psychologically controlling through overt verbal attacks and subtle judging comments. Jackie says:

We still fight almost everyday because he does not like my job as a TSW. It makes him jealous, I guess. Sometimes he yells at me, calling me a “cheap duck, a chimpanzee she male” because I sell my body for sex. The yelling and insults happen all the time. Still, I don’t want to leave him because it is not easy to find a partner in the transgender community. He often says I am indecent, I am a slut, and he does not want me to be a sex worker. But he cannot provide a solution for our financial situation. He calls me a sissy and an ass worker. I would love to find a man who wants me and accepts me for who I am.

Pan (38) experienced the same problem as well. She says:

My partner said I am unattractive, provincial, and look like a giant chimpanzee. He says I look unnatural. His words really hurt and make me seriously question myself “do I really look like a giant chimpanzee she male?” He always says I am such a dirty woman, while he himself is clean, neat, and tidy. I think I lack the confidence to make a firm decision to leave him. I have been tolerating him lately, but I am suffering from anxiety and insomnia. I lie awake in bed and think about the housework that I might have forgotten to finish, as this would irritate him…

(Pan, 38)

#### 4.2.4. Financially Ripped Off

Shenshen (42) is a TSW who has been working in this field for three years. She was so passionate about the topic that she brought her photo album to the interview. As she displayed the images of her boyfriend, she admitted that, at first, she enjoyed a very sweet and intimate life with him. But three months later, the honeymoon was over. Shenshen said this memory is her only fantasy about romance. The reality was that this man only wanted her money. Shenshen (42) says:

… We had fights and he would beat me. Afterward, he would beg me to forgive him. He never had money for rent, bills, not even for drugs. I supplied everything! He asked me for a six-month allowance of 10,000 yuan (US $1400). I thought about it, and finally decided to give it to him. My hope was that if he could appreciate me, perhaps we could get back to being a normal couple. But I was wrong. He took the money and forgot about me. Now I just want him out of my life.

Wen (45) shared another story of deceit:

My partner will try every excuse to ask for money. He said his parents needed money for surgery, or he needed money for his new business, or his brother needed money to go to college, or his relatives needed money to repair their house. I know they are all lies, but if I had any spare money, I would have given it to him. But I think it is unhealthy and unfair to me, so finally I yelled and asked him to leave. It was a painful decision. Living with him was one rip off after another. I am still struggling with him…

Qiang (33) finally saw the truth when she recorded her boyfriend’s drunk and abusive taunts one night and played them for the investigator:

Don’t you dare think you are a virgin or a princess! Of course, I just live with you for the money. You think someone will love you? A monster? Hello! Wake up monster! I found out you are broke and mean. So, I am leaving to find another big fish—of course, I want to dump you away! You think it is easy to live with you? Never! I have to tolerate a lot of pain to be with you. Don’t ever think that living with a monster, a she male, is fun, huh?! I just want expense money, food and rent from you. You should be grateful I haven’t stabbed you…

#### 4.2.5. Stalking and Extortion

While the interviewees confirmed they had broken up with the partners described throughout this paper, many of these relationships were still unresolved. Stalking and extortion were identified as some of the situations they had to deal with. Fafa (29) said her partner continued to harass her for a full year after they broke up,

I would get weekly deliveries to my apartment of packages containing urine, shit, or semen. I moved four times to escape him. He also threatened to send naked pictures of me to my mother and post them on WeChat, TikTok, and Weibo. Once I gave him 10,000 yuan (US $1538) to leave me alone and he agreed. But three months later he demanded more money for drugs, and I gave him another 10,000 yuan (US $1538). Then he came back a third time, this time demanding 50,000 yuan (US $7692). This much, I could not afford... So, he sent naked photos of me to my mother, my siblings, and even to people in my village… right now everyone knows I am a transgender sex worker… [sighs], I guess I am famous now.

Hui (38) also experienced public humiliation from a vindictive ex-lover:

I broke up with my partner one month ago. For revenge, he sent naked photos of me to my sister and mother, complete with my female top and male bottom. He also sent pictures by mail to the village mayor. So, everyone knows I am a transgender who works in the bushes selling sex. They are so ashamed of me. They cut all ties with me and I am now dead to the family. I heard my father had a stroke, and my mother was denounced by the villagers who blamed her for giving birth to a monster. My family is banned from the ancestral halls as I am dirty, sinful, and accursed.

## 5. Sisterhood and Informal Social Networks

The interviewees all agreed they could not rely on the police or social policy makers for help. The TSWs learned to first seek help from other transgender sex workers. This informal network was described as a ‘sisterhood’ of support. For example, Xing (31) said she once asked for police help but they said the problem was of her own making:

After we broke up, my ex-boyfriend continued stalking me on WeChat and TikTok. I called the police and complained to them. They came to my apartment, and said: “You know your identity, right? Did you ever have your new identification card with your new gender? If you cannot provide this evidence, please do not waste our time. You transgenders affect our city image! This is not police business, go find your own way, clean up your own mess.” Around 3:00 a.m. I was so depressed I was going crazy. I called my sister and talked to her about what happened. She was so kind and nice. She talked to me for nearly 3 h. Otherwise, I might have committed suicide…

(Xing, 31)

Fan (24) said her sisters help her feel like a normal girl living a normal life:

My sister from Beijing occasionally visits me twice per month; she usually brings my favorite desserts and we go shopping for cosmetic products. Sometimes, we would go to a salon or beauty parlor just to enjoy ourselves. Shopping is really a therapy, and her visits help me to enjoy life and find meaning. Sometimes, we would organize our own little party and invite our sisters to come join us. Last time was Christmas, and we had a great time. Her support is important to help me release my emotion…

From the conversations of the TSWs, we can see that advice and support come from their sisterhood. However, these were not a ‘tight circle of friends’ to most of the TSWs. They are friends who hang out together all the time to ask for help and personal counselling. The sisters are co-workers, colleagues, friends, and sometimes even one-time lovers. From their shared experience as TSWs and similar struggles, they lean on one another for advice, support, and sometimes escapist fun. Juan (32) told the author that she finds strength just by talking to her sisters about her experiences. She says:

When I am alone and feel depressed about being bullied by my partner, I can text my ‘sister’ and she will stop by for a chat. I feel like I finally have a shoulder to cry on. She doesn’t judge, and I can trust her. I can talk to her about my partner and she will listen before giving advice. Sometimes she says I should be patient or forgiving. Sometimes she says my partner is taking advantage of me and I should leave the jerk immediately…

Yoyo (44) had an experience similar to Meiha (48), she says:

I cannot ask the police to protect me if my partner keeps harassing or stalking me. The only other person I will talk to for help is my sister or someone at the NGO. I remember my sisters and I once vowed to help each other improve our lives. For example, when I sell sex in the public park I will ask the sisters nearby to keep watch for me. If the police show up, they can quickly call a gangster to our location to interrupt the arrest… or even go with me in solidarity to the police station… my sisters are my shelter… Police have put me in detention twice for selling sex in the park...

Yee (24) said the NGOs provide a shelter, a place for her to heal. Especially if a staff member is trans, it becomes a safe space to meet people and talk when she feels unhappy. She says:

I will go to the NGOs to find a staff to chat with if I have a problem. I think the ones at the NGO who understand me the best are gay or trans. I hope they will one day provide a qualified counsellor or social worker to help us. Since I came out to my family I have had a horrible relationship with them. I don’t have many close friends and they definitely won’t accept me as a transgender...

Perhaps the most important function of the sisterhood is to provide a reality check and to help them feel ‘normal.’ The TSW community is relatively small and vulnerable; they do not make friends easily as they have found most people have judged them as offensive and disgusting, immoral monsters. Neither male nor female, and giving their bodies in sex-for-money transactions, makes things even worse. Since the partner-relationships are fraught with verbal and physical violence, TSWs rely on each other to be able to let down their guard. They actively arrange time with other sisters to accompany them for running errands, shopping, and enjoying the little pleasures in life, like going out for a dessert. The sisters may gossip and criticize each other, but in a life full of danger, struggle and mistrust, they learn to trust each other, knowing they are each on the same trans-journey in life.

## 6. Discussion and Conclusions

### 6.1. Support from Sisterhood/Informal Network

The home environment for TSWs may be as unsafe as the work environment. The 25 TSWs interviewed offered evidence of their partners routinely attacking them with a steady barrage of homophobic, sexist and derogatory comments. When the home environment is full of intimidation and fear, the TSWs become silenced because their identity is not legitimate in China. Government and media outlets have long discriminated against LGBT groups. These include the representatives of SAPPRFT (State Administration of Press, Publication, Radio, Film and Television of The People’s Republic of China), local TV stations, newspapers, and even internet platforms like Sina online. The Chinese government strategically represses transgender communities in order to keep them silent and invisible. Among the interviewees, 14 out of 25 TSWs had experience being sent to a detention center for publicly engaging in immoral acts. The 25 TSWs are judged to be unworthy victims because they transgress cultural norms by acting seductively, being manipulative and provocative, and having ambiguous relationships with men. Therefore, police routinely target TSWs as they receive complaints from nearby residents to public parks.

The informal sisterhood network remains hidden and safe from the judging, criminalizing, and stigmatizing gaze of the public. They use mobile phone and chat functions to help develop a sense of intimacy and trust among themselves. The sisters offer assistance, support, and care, which helps them to subsist in a hostile environment. In addition, due to the complexities of shame, isolation, and the stigma of gender identity and mental illness, the TSWs rely heavily on the Internet to access resources in a safe, non-judgmental environment. TSWs have also learned to mobilize their communities and work together when dealing with the police. Being in groups is the best way to bolster the confidence of the TSWs in advocating for themselves and minimizing the effects of stigma, violence, and arrests from the police.

The help from their sisterhood seldom involves money or political lobbying. Mostly they go to each other for psychological and emotional comfort. These links are relational more than transactional. The perspective of queer criminology argues that criminal justice and social policies can help to solve the problems faced by TSWs. However, for today’s TSWs in China, this will not become a reality anytime soon. For example, Chinese LGBT (lesbian, gay, bisexual and transgender people) held an online protest against Sina Weibo on 13 April 2018. Sina Weibo had banned online content “related to homosexuality” that day. The success of the Chinese LGBT group made an unprecedented step in the battle against widespread discrimination, which further forced the Chinese authorities to react to guarantee de-criminalization and de-stigmatization of homosexuality in Chinese society [44]. This shows the importance of an informal network and how TSWs can also develop online communities to build cohesion and trust.

### 6.2. No Law Protection and Criminal Justice

According to Chinese law, the archetypical definition of sexual violence is “forced penetration of a victim through violence or intimidation” [45]. The law usually protects heterosexual women who experience sexual harassment and violence by heterosexual men. Although sexual harassment was included in the “Law to Protect Women’s Rights” in 2005, it provided no legal definition of, or punishment for, the act, only stipulating the nature of sexual harassment as a civil affair, rather than a criminal violation [46]. For TSWs, the law does not extend to men who transform to women, or even identify as women. There is no specific Chinese government law which protects against violence or fraud at the hands of a transgender male’s intimate partner. The Chinese government policies and documents largely define TSWs in terms of abnormality, and ‘not encouraging, not discouraging and not promoting,’ which reflects the views of laws, regulations and media [47].

## 7. Conclusions

The 25 participants in this study admitted they struggle with several types of IPV. Sometimes the intimate partner violence experienced by the 25 transgender sex workers is mild, but sometimes it is life-threatening. All of them suffered some form of sexual violence, and over 15 out of 25 TSWs suffered from more than one type of IPV mentioned in this article. This article affirms the queer criminology position that gender is non-binary, and those who are biologically men can be culturally identified as women. However, existing aspects of criminal justice and the legal system do not help TSWs. The psychological impact of IPV is marked by depression, anxiety, post-traumatic stress disorder, depression, anxiety and even physiological effects like migraines [48,49,50]. The participants in this study agreed that there will not be any groundbreaking policy to protect TSWs by the China government anytime soon. Although countries like the United States have enacted laws in 2020 to protect transgenders from discrimination in the workplace, the first step for China is to simply acknowledge that transgenders exist and deserve the same protections as everyone else. TSWs have tried to find informal networks like talking to their sisters, meeting friends on the Internet, and looking for help through NGOs. Some NGOs provide counselling and rest areas for TSWs if they suffer IPV. Therefore, the informal networks of TSWs constitute a challenge to the Chinese government.

By including TSWs as homosexual “queered” objects, this article makes significant contributions for policy makers, state actors, NGOs and other social service providers. TSWs should not only rely on their informal network to help, but formal avenues may also include court orders or police reporting. This article proposes that government and police begin to provide friendly policies towards TSWs, such as a professional counselling service, job opportunities, and even a retraining scheme. TSWs are, in their own way, taking a page from the American Black Lives Matter movement in June 2020. TSWs are shouting “We Exist! We Want Freedom! We deserve the same rights enjoyed by everyone else in China!” Now it is time for China to listen.

This article helps us understand TSWs in at least three ways. First, various and specific forms of IPV have been described by TSWs. Second, these IPV episodes are presented against socio-cultural structures which frame the interactions between TSW and their partners. Third, there is an informal network of TSWs which enables them to help each other in times of need. The experiences of these TSWs reveal how they respond to violence in their day-to-day lives, affirming their precarious existence. They are socially, economically, politically and legally marginalized; they struggle financially and emotionally in the face of sexual violence. Some limitations of these findings include the sample, which only included sex workers who are biologically born as male but culturally identify as female. Having a more diversified sample of the transgender population can help focus future research. Further research should also compare transgenders who experience IPV with those who have not to provide insights regarding psychological stress, coping effectiveness, and the efficacy of self-help programs.
